# Reliability of a retrospective decade‐based life‐course alcohol consumption questionnaire administered in later life

**DOI:** 10.1111/add.13012

**Published:** 2015-07-14

**Authors:** Steven Bell, Annie Britton

**Affiliations:** ^1^ Research Department of Epidemiology and Public HealthUniversity College LondonLondonUK

**Keywords:** Alcohol, blood pressure, cross‐sectional cohort, life‐course, longitudinal, measurement, reliability, AUDIT‐C, retrospective

## Abstract

**Background and aims:**

Retrospective measures of alcohol intake are becoming increasingly popular; however, the reliability of such measures remains uncertain. This study assessed the reliability of a retrospective decade‐based life‐course alcohol consumption questionnaire, based on the standardized Alcohol Use Disorder Identification Test–Consumption (AUDIT‐C) administered in older age in a well‐characterized cohort study.

**Design, setting, participants and measurements:**

A retrospective alcohol life‐grid was administered to 5980 participants (72% male, mean age 70 years) in the Whitehall II study covering frequency of drinking, number of drinks in a typical drinking day and frequency of consuming six or more drinks in a single drinking occasion in the teens (16–19 years) through to the 80s. A subsample of 385 individuals completed a repeat survey to determine test–retest reliability. Retrospective measures were also compared with prospectively ascertained information and used to predict objectively measured systolic blood pressure to test their predictive validity.

**Findings:**

Across all decades of life, test–retest reliability was generally good (κ range = 0.62–0.78 for frequency, 0.55–0.62 for usual number of drinks and 0.57–0.65 for frequency of consuming six or more drinks in a single occasion). The concordance between prospective and retrospective measures was consistently moderate to high. The life‐grid method performed better than a single question in identifying life‐time abstainers. Retrospective measures were also related to systolic blood pressure in the manner anticipated.

**Conclusion:**

A retrospective decade‐based AUDIT‐C grid administered in older age provides a relatively reliable measure of alcohol consumption across the life‐course.

## Introduction

The effect of variation in alcohol consumption across the life‐course is an important yet neglected topic 1, 2, 3, 4, 5, 6. Clearly, ascertaining information regarding alcohol consumption at a single point in time is an inadequate measure of cumulative exposure to alcohol across the life‐course 6. It is fundamental that repeat measures of alcohol consumption are used to capture fluctuations in drinking habits across the life‐course (as well as to minimize the error associated with drinking category misclassification 7, 8, 9) that may be important in predicting both morbidity and mortality 1, 2, 4, 10, 11, 12. However, this is difficult in practice. Large‐scale prospective longitudinal studies are required, and these can be both costly and time‐consuming (especially for outcomes that have relatively large latency periods; e.g. wanting to explore whether alcohol trajectories from late adolescence to early adulthood are associated with cardiovascular disease, which manifests typically much later in the life‐course). It is therefore important that less resource‐intensive options are explored and their reliability assessed.

One approach is to construct retrospective accounts of an individual's life‐time drinking history. Previous work on retrospective measures of alcohol consumption has shown that they are relatively reliable in capturing life‐course patterns of consumption 2, 13, 14, 15, 16. However, the majority of retrospective‐based measures of drinking behaviour [e.g. the Lifetime Drinking History (LDH) questionnaire] are time‐consuming, and therefore not appropriate to use in large‐scale general population studies 2, 17, 18.

Decade‐based retrospective measures of consumption have been advocated as a feasible alternative to in‐depth retrospective measures 2—having reasonably good predictive validity for alcohol use disorders as well as diabetes and coronary problems 16, 17. However, these studies have typically lacked prospective data from participants on their drinking habits prior to the collection of retrospective measures as a means of validating participants’ recall.

Previous discussion on the topic of assessing alcohol consumption across the life‐course with retrospective measures has highlighted the issue that often asking participants to recall exact amounts is typically associated with poorer recall, while asking them to provide relative rankings seems to produce stable responses 18. This supports the idea of assessing life‐time consumption using a short standardiszed tool, such as the ‘consumption’ component of the Alcohol Use Disorders Identification Test (AUDIT‐C), which provides specific response categories for an individual to choose from. In the same discussion 18, several other factors were highlighted which further reinforce the use of a simple to administrate tool when assessing life‐time alcohol consumption. First, the level of detail that one requests participants to recall is important (providing participants with three questions with fixed responses, as in the AUDIT‐C, is less burdensome than procedures which require participants to construct their own spline‐based drinking trajectories). Secondly, the primary purpose of retrospective measures are to gain a general understanding of an individual's drinking history, therefore explicitly detailed accounts are not required; in fact, it has been argued that retrospective measures should be kept as simple as possible and this might actually lead to more accurate retrospective accounts of life‐course consumption 18.

However, while the focus thus far has been on the positives of retrospective measures, it is important to note that they are inherently limited 2, and prone to several major sources of bias. These include, but are not limited to, the extent to which current characteristics (such as age, gender, socio‐economic position and present drinking habits) influence recall and how inconsistent responses are addressed 2, 18. A particularly important subtopic under the umbrella of inconsistency in responses over time is the issue of life‐time abstainers 7, 8, 9, 19. For example, in a British birth cohort study, 67% of self‐reported life‐time abstainers (at age 45 years) reported alcohol consumption at an earlier measurement occasion, and only 53% of these individuals reported being never drinkers only 3 years earlier 19. One might imagine that retrospective measures of life‐course consumption obtained in later life would be even more susceptible to this bias.

The purpose of this paper is therefore to assess the reliability of a retrospective decade‐based life‐course alcohol consumption questionnaire, based on the AUDIT‐C, administered in older age.

## Methods

### Study sample

The Whitehall II prospective cohort study started as a sample of 10 308 British civil servants (6895 men and 3413 women) based in London offices who were aged 35–56 years at entry into the study in 1985–88 20. It was established to examine the social gradient in health and disease in both men and women, specifically by exploring the pathways and mechanisms through which social position influences health. Participants have been followed‐up regularly ever since through a combination of clinical examinations and self‐administered questionnaires. The current investigation makes use of prospective data on alcohol consumption collected at multiple times from baseline onwards in a sample of participants who took part in the most recent phase (phase 11, 2012–13) who completed a retrospective life‐course alcohol grid (described below; *n* = 5980, 58% of the original sample and 95% of those participating in the most recent phase). A subsample of participants completed a repeat alcohol life‐course survey (*n* = 385). The subsample consisted of a random‐stratified sample (weighted by age group and gender proportions) of the initial 3000 responders at phase 11. In total, 500 repeat questionnaires were posted to participants (400 to those who attended the clinical visit and 100 to those who required a home visit), with a final response rate of 77%. The Whitehall II study is approved by the Joint University College London/University College London Hospital Committees on the Ethics of Human Research (Committee Alpha). All participants provided written informed consent.

Whitehall II data, protocols and other metadata are available to bona fide researchers for research purposes. Please refer to the Whitehall II data‐sharing policy at http://www.ucl.ac.uk/whitehallII/data‐sharing.

## Assessment of alcohol consumption

### Prospective measures of consumption from baseline

#### Frequency of consumption

Participants were asked to report the frequency that they consumed alcohol in the past 12 months seven times during follow‐up [at phases 1 (baseline) (1985–88), 2 (1989–90), 3 (1991–94), 5 (1997–99), 7 (2002–04), 9 (2007–09) and 11 (2012–13)]. Response options were: never, special occasions, monthly, weekly, daily or more than once daily.

As participants could report information concerning their frequency of consumption at multiple times during the course of a specific decade of their life, the modal frequency response reported by participants while they belonged to specific decade‐based age‐groups (e.g. aged 40–49 years, 50–59 years) during follow‐up was taken as an indicator of how often they consumed alcohol while that age.

#### Usual number of drinks in a single drinking session

The first two study phases contained questions on the usual number of drinks (fixed responses of: none, one to two, three to four and five or more drinks) a participant consumed in a single drinking session separately for beer and for wine and spirits combined. As these questions did not allow for participants to report drinking sessions whereby they mixed beer with wine/spirits, the highest frequency response to either item was taken as a participant's usual amount (e.g. someone reporting consuming 1–2 pints of beer but five or more glasses of wine/spirits would be assigned to the five or more drinks category). Again, the modal response to this question while participants belonged to specific decades was taken as their usual amount at that age.

#### Frequency of drinking six or more drinks

Unfortunately, no prospective information regarding frequency of consuming six or more drinks in a single occasion was available throughout Whitehall II follow‐up.

#### Life‐time abstention

We used prospectively collected information relating to both frequency of alcohol consumption in the past 12 months collected at each study phase, as well as responses to a question asking those who reported no consumption in the past year as to whether they were always a non‐drinker, which was introduced at phase 3. We used a strict definition of life‐time abstention, insisting that participants consistently reported no alcohol consumption 21. This variable was updated throughout follow‐up to examine misclassification bias 22.

### Retrospective alcohol life‐course grid

Life‐course alcohol consumption was defined using decade‐based grids starting with information in the teens (16–19 years) and spanning to the 80s (and older) on the three components of the AUDIT‐C questionnaire: frequency of consumption, number of drinks on a typical drinking day and frequency of consuming six or more drinks at a single occasion 23, 24, 25, 26.

### Other variables

#### Demographics

The age of participants at each phase was calculated and used to define the decade of life to which they belonged. Gender was recorded at baseline. Socio‐economic position was defined, using either current or last recorded civil service employment grade. Civil service grades are hierarchical and are based on salary and work role; we defined socio‐economic position in three levels as high (unified grades 1–7), intermediate (executive officers) or low (clerical or support staff), as described previously 27, 28. Participants were also asked about their current smoking habits and defined as current‐, ex‐ or never smokers.

#### Current drinking habits

Information regarding current drinking habits was obtained via the standard AUDIT‐C questionnaire, covering drinking frequency in the past 12 months as well as usual amount consumed in a drinking session and frequency of consuming six or more drinks in the same period. Participants were defined as current hazardous drinkers using their AUDIT‐C scores at a threshold of scores of four for men and three for women 26.

#### Memory

Participants completed the 30‐item Mini Mental State Examination (MMSE). A threshold of scores of less than 27 was used to identify individuals with current mild cognitive impairment 29.

#### Objective health outcome

Systolic blood pressure (SBP) was measured with participants in the sitting position. SBP was determined twice, with 5 minutes rest between measurements—the average of these two readings was used as the final measure.

## Analytical plan

### Test–retest

Weighted kappa coefficients (κ) were used to assess the test–retest reliability of individual life‐course AUDIT‐C items. Weighted kappas were chosen as they are preferable when assessing the agreement between ordinal scales 30, 31 as they allow for deviations on categorical responses to be weighted according to the distance of digression between options. Linear weightings were applied 32, 33, 34. Several definitions/cut‐points exist for strength of agreement 31, 35, 36; generally, values of 0.41 or above are seen as acceptable while coefficients greater than 0.61 are interpreted as good.

#### Retrospective recall versus prospective measures

Polychoric correlations (useful when comparing two ordinal variables which are assumed to measure the same underlying latent construct) 37, 38 were estimated between retrospective decade‐based measures of frequency of consumption/usual number of drinks on one occasion and average decade‐based self‐reported measures which were obtained prospectively from age 35 onwards (participants were aged 35–55 at baseline). Spearman's rho (ρ) was also estimated.

#### Predicting objective health outcome

Linear regression was used to estimate differences in SBP on the basis of current reports of consuming six or more drinks in a single drinking occasion at least monthly as well as retrospectively assessed consumption with adjustment for current age, gender, socio‐economic position and smoking status. SBP was chosen as an outcome based on its causal association with alcohol consumption 39, as well as previous studies using decade‐based measures to predict it 17.

All analyses were conducted using Stata version 13 (StataCorp, TX, USA) 40.

## Results

### Descriptive statistics

Presented in Table 1 are descriptive statistics for the analytical sample. The mean age of participants was 70 years. The majority of the sample were male, high to intermediate socio‐economic position and non‐smokers. Average weekly alcohol intake was 10 UK units in the pooled sample. Drinking on a weekly basis was the most common drinking frequency. Few participants reported consuming more than the recommended UK drinking guidelines in a usual drinking day (3/4 units for women and men, respectively). Drinking more than six drinks in a single drinking episode was also uncommon.

**Table 1:** Characteristics of individuals from the Whitehall II study who completed the alcohol life‐grid questionnaire at study phase 11.

	*n*	% *or mean* (*SD*)
Age	5980	69.7 (5.8)
Sex		
Men	4291	71.8
Women	1689	28.2
Socio‐economic position		
High	1908	31.9
Intermediate	2509	42.0
Low	1563	26.1
Smoking status		
Never	2521	44.4
Ex	2941	51.8
Current	215	3.8
Current drinking habits		
UK units in past week	5906	10.0 (11.3)
Drinking frequency (12 months)		
Never	167	2.9
Once a month or less	767	13.4
2–4 times per month	1005	17.5
2–3 times per week	1465	25.5
4+ times per week	2332	40.7
Number of drinks per drinking day (12 months)		
1–2 drinks	4112	73.8
3–4 drinks	1205	21.6
5–6 drinks	206	3.7
7–9 drinks	37	0.7
10+ drinks	10	0.2
How often consumed 6+ drinks in a single session (12 months)		
Never	4244	74.8
Less than monthly	907	16.0
Monthly	272	4.8
Weekly	206	3.6
Daily/almost daily	45	0.8

SD = standard deviation.

### Test–retest reliability

#### Frequency of consumption

Kappa coefficients relating to frequency of alcohol consumption are presented in Table 2. Within the whole sample, across all occasions, reliability was generally good (κ = 0.62–0.78, increasing chronologically).

**Table 2:** Kappa coefficients for retrospective AUDIT‐C items across the life‐course, by age, gender, socio‐economic position, current drinking and presence of mild cognitive impairment.

	*Age 16–19*		*Age 20–29*		*Age 30–39*		*Age 40–49*		*Age 50–59*		*Age 60–69*		*Age 70–79*	
	*n*	*κ* a	*n*	*κ*	*n*	*κ*	*n*	*κ*	*n*	*κ*	*n*	*κ*	*n*	*κ*
How often did you have a drink containing alcohol?														
Whole sample	374	0.62	380	0.63	382	0.70	382	0.67	382	0.71	370	0.78	161	0.73
Age														
50/60s	207	0.64	208	0.57	209	0.70	209	0.72	209	0.74	197	0.83	–	–
70+	167	0.57	172	0.67	173	0.61	173	0.61	173	0.67	173	0.72	–	–
Gender														
Male	256	0.57	260	0.57	261	0.65	261	0.67	261	0.71	257	0.79	107	0.74
Female	118	0.66	120	0.69	121	0.67	121	0.67	121	0.70	113	0.76	54	0.69
SEP														
High	114	0.52	116	0.58	117	0.64	117	0.63	117	0.58	113	0.72	49	0.71
Intermediate	161	0.65	164	0.63	164	0.60	164	0.63	164	0.72	161	0.77	65	0.65
Low	99	0.64	100	0.62	101	0.76	101	0.74	101	0.76	96	0.81	47	0.79
Current drinking														
Non‐hazardous	203	0.60	207	0.65	209	0.69	209	0.67	209	0.66	204	0.71	94	0.56
Hazardous	171	0.63	173	0.57	173	0.57	173	0.52	173	0.49	166	0.57	67	0.57
Mild cognitive impairment														
No	322	0.64	327	0.61	328	0.66	329	0.67	329	0.71	316	0.79	130	0.72
Yes	28	0.53	27	0.78	28	0.76	27	0.83	27	0.66	28	0.71	17	0.67
How many drinks containing alcohol did you have on a typical day when you were drinking?														
Whole sample	373	0.55	381	0.58	379	0.56	377	0.58	377	0.59	367	0.62	155	0.56
Age														
50/60s	205	0.55	207	0.58	207	0.56	207	0.60	208	0.64	196	0.64	–	–
70+	168	0.51	174	0.54	172	0.53	170	0.53	169	0.50	171	0.58	–	–
Gender														
Male	255	0.51	259	0.57	259	0.55	258	0.58	256	0.59	252	0.62	104	0.58
Female	118	0.60	122	0.54	120	0.53	119	0.53	121	0.53	115	0.59	51	0.49
SEP														
High	115	0.52	117	0.60	117	0.47	116	0.61	116	0.64	113	0.70	47	0.51
Intermediate	162	0.55	164	0.56	164	0.58	163	0.55	164	0.57	161	0.59	64	0.58
Low	96	0.57	100	0.58	98	0.61	98	0.56	97	0.53	93	0.55	44	0.58
Current drinking														
Non‐hazardous	203	0.55	209	0.64	207	0.59	206	0.57	206	0.48	201	0.51	89	0.46
Hazardous	170	0.54	172	0.50	172	0.49	171	0.54	171	0.58	166	0.58	66	0.53
Mild cognitive impairment														
No	321	0.53	327	0.58	326	0.52	325	0.55	324	0.58	314	0.66	126	0.50
Yes	28	0.68	29	0.50	28	0.75	28	0.74	28	0.60	29	0.46	16	0.52
How often did you have six or more drinks on one occasion?														
Whole sample	379	0.63	381	0.64	378	0.65	379	0.61	379	0.59	365	0.62	159	0.57
Age														
50/60s	208	0.65	207	0.67	205	0.68	206	0.64	206	0.59	192	0.62	–	–
70+	171	0.54	174	0.57	173	0.58	173	0.57	173	0.59	173	0.62	–	–
Gender														
Male	258	0.60	259	0.60	259	0.64	257	0.63	257	0.62	251	0.64	105	0.60
Female	121	0.72	122	0.73	119	0.62	122	0.52	122	0.45	114	0.51	54	0.32
SEP														
High	115	0.61	117	0.60	116	0.64	115	0.67	115	0.66	111	0.62	47	0.69
Intermediate	164	0.63	163	0.69	162	0.65	163	0.59	163	0.57	159	0.66	65	0.46
Low	100	0.64	101	0.58	100	0.62	101	0.56	101	0.50	95	0.53	47	0.34
Current drinking														
Non‐hazardous	209	0.62	210	0.69	210	0.56	208	0.47	208	0.35	203	0.26	93	–0.03
Hazardous	170	0.63	171	0.59	168	0.67	171	0.65	171	0.62	162	0.64	66	0.60
Mild cognitive impairment														
No	325	0.63	326	0.63	324	0.63	325	0.61	325	0.61	311	0.61	130	0.52
Yes	29	0.68	29	0.68	28	0.57	29	0.48	29	0.31	29	0.49	17	–0.10

a Linear weightings applied. SEP = socio‐economic position; AUDIT‐C = Alcohol Use Disorder Identification Test–Consumption.

Men had consistently lower reliability for early life measures (ages 16–29) than women, but from midlife onwards gender coefficients were relatively equal. Binary categorization of age into 50–69 and 70+ years revealed that reliability was typically poorer in older participants. Recall was also generally poorer in participants who belonged to the highest socio‐economic group. Present hazardous drinkers had lower reliability in their recall, while no consistent difference in reliability was observed for those with mild cognitive impairment.

#### Usual number of drinks on a drinking day

Test–retest reliability for the usual number of drinks on a typical drinking day (Table 2) was generally lower than that observed for frequency of consumption. However, in the combined sample reliability estimates remained within acceptable ranges (κ = 0.55–0.62).

No consistent gender bias was observed for the reliability of usual number of drinks recalled across the life‐course [with the exception that in those aged 70–79, women had lower (κ = 0.49) scores than men (κ = 0.58)]. Throughout recall of early life alcohol consumption no stable age‐related bias was observed; however, from mid‐life (40+ years) onwards those in the older age group consistently had lower κ values than younger cohort members. Those from the highest socio‐economic group tended to recall more clearly the number of drinks they reported consuming in a typical drinking day than those from lower socio‐economic groups, as did current non‐hazardous drinkers. Again, no notable pattern was observed between those with mild cognitive impairment and those without.

#### Frequency of consuming six or more drinks in a single occasion

Reliability estimates for frequency of consuming six or more drinks in a single occasion (Table 2) generally exceeded those observed for the usual number of drinks on a drinking day, and were similar to those estimated for frequency of consumption when looking earlier in the life‐course. In the whole sample, reliability coefficients fell consistently within acceptable ranges (κ = 0.57–0.65).

Women had higher reliability in their recall from age 16–29, but from age 40 onwards men were more consistent in their recall. Younger participants were also more consistent with their recall of frequency of consuming six or more drinks in a single occasion up until the age of 50, at which point reliability estimates were similar to older participants. Those from the lowest socio‐economic group were less consistent with their recall, as were those whose drinking pattern was considered non‐hazardous based on their AUDIT‐C score. In this instance, those with mild cognitive impairment had lower κ values than those without.

### Retrospective recall versus prospective measures

#### Frequency of consumption

Correlations between prospectively collected data on frequency of consumption with retrospectively collected information are presented in Table 3. Both polychoric correlations and Spearman's rho coefficients were consistently high and increasing in magnitude from age 30 onwards in the whole sample.

**Table 3:** Polychoric correlations and Spearman's rho between retrospective frequency of drinking and average amount consumed with prospective measures of the same construct.

	*Age 30–39*			*Age 40–49*			*Age 50–59*			*Age 60–69*			*Age 70–79*		
	*n*	*Poly*	*Rho* †	*n*	*Poly*	*Rho*	*n*	*Poly*	*Rho*	*n*	*Poly*	*Rho*	*n*	*Poly*	*Rho*
Frequency															
Whole sample	1853	0.70	0.62	4667	0.73	0.64	5688	0.80	0.72	4511	0.85	0.77	1479	0.85	0.80
Age															
50/60s	–	–	–	3299	0.73	0.63	3189	0.84	0.74	2037	0.89	0.80	–	–	–
70+	–	–	–	1368	0.72	0.65	2499	0.76	0.68	2474	0.82	0.74	–	–	–
Gender															
Male	1352	0.68	0.61	3387	0.70	0.61	4103	0.79	0.68	3279	0.83	0.72	1058	0.83	0.76
Female	501	0.70	0.62	1280	0.74	0.68	1585	0.81	0.76	1232	0.90	0.81	421	0.86	0.81
SEP															
High	630	0.65	0.54	1507	0.68	0.57	1839	0.76	0.63	1420	0.85	0.71	476	0.84	0.73
Intermediate	842	0.71	0.64	2009	0.73	0.65	2395	0.82	0.74	1855	0.82	0.74	568	0.82	0.77
Low	381	0.68	0.61	1151	0.72	0.65	1454	0.78	0.72	1236	0.85	0.79	435	0.86	0.81
Current drinking															
Non‐hazardous	769	0.67	0.59	2076	0.69	0.62	2631	0.72	0.65	2128	0.73	0.66	779	0.75	0.68
Hazardous	1084	0.56	0.47	2591	0.52	0.43	3057	0.59	0.46	2383	0.65	0.49	700	0.51	0.40
Mild cognitive impairment															
No	1615	0.69	0.61	3855	0.72	0.63	4544	0.81	0.72	3519	0.85	0.76	1027	0.86	0.80
Yes	92	0.81	0.74	370	0.77	0.70	564	0.76	0.68	510	0.82	0.75	251	0.82	0.77
Average amount															
Whole sample	1833	0.54	0.45	3824	0.54	0.44	1720	0.57	0.44	–	–	–	–	–	‐‐
Age															
50/60s	–	–	–	2487	0.54	0.44	–	–	–	–	–	–	–	–	‐‐
70+	–	–	–	1337	0.55	0.44	–	–	–	–	–	–	–	–	‐‐
Gender															
Male	1341	0.54	0.44	2810	0.53	0.44	1228	0.60	0.46	–	–	–	–	–	‐‐
Female	492	0.52	0.46	1014	0.54	0.42	492	0.46	0.35	–	–	–	–	–	‐‐
SEP															
High	622	0.48	0.38	1211	0.58	0.46	563	0.65	0.50	–	–	–	–	–	‐‐
Intermediate	837	0.56	0.38	1632	0.53	0.44	668	0.56	0.43	–	–	–	–	–	‐‐
Low	374	0.58	0.50	981	0.52	0.44	489	0.50	0.37	–	–	–	–	–	‐‐
Current drinking															
Non‐hazardous	755	0.50	0.40	1709	0.57	0.43	878	0.52	0.38	–	–	–	–	–	‐‐
Hazardous	1078	0.50	0.42	2115	0.44	0.38	842	0.55	0.43	–	–	–	–	–	‐‐
Mild cognitive impairment															
No	1600	0.57	0.46	3119	0.54	0.44	1214	0.54	0.41	–	–	–	–	–	‐‐
Yes	91	0.59	0.43	318	0.56	0.44	273	0.62	0.48	–	–	–	–	–	‐‐

Poly = polychoric correlation;

† Spearman's rho; SEP = socio‐economic position.

No significant age, gender, socio‐economic position or memory related biases were observed. Those whose current drinking was defined as hazardous tended to have poorer agreement between prospective and retrospectively recalled information.

#### Number of drinks on drinking day

As in the test–retest analyses, generally the correlations between retrospective measures of number of drinks consumed on a typical drinking day and prospective measures relating to the same item were lower than those found for measures relating to frequency (Table 3).

In the whole sample, correlations tended to remain stable over age (from 35 years onwards). The correlation between retrospective and prospective measures was greater in men at age 50–59 years than in women. No consistent difference between socio‐economic groups was observed, nor by current drinking status or presence of mild cognitive impairment.

#### Life‐time abstainers

There were inconsistent reports of life‐time abstention by participants in the same questionnaire (Tables 4 and 5). For example, among the 94 participants who answered ‘yes’ to the question: ‘Have you always been a non‐drinker?’ we found that 42 of them provided a response on the alcohol life‐course grid that was consistent with this. When we examined data from earlier phases, only 12 of these individuals actually met the definition of life‐time abstention (Table 5).

**Table 4:** Life‐time non‐drinking status by method of ascertainment among self‐identified non‐drinkers at phase 11 [*n* (%)].

	*Pooled* (*n* = *374*)		*Men* (*n* = *183*)		*Women* (*n* = *191*)	
	*Yes*	*No*	*Yes*	*No*	*Yes*	*No*
Always a non‐drinker	94 (25.1)	280 (74.9)	38 (20.8)	145 (79.2)	56 (29.3)	135 (70.7)
Life‐grid non‐drinker	49 (13.1)	325 (86.9)	15 (8.2)	168 (91.8)	34 (17.8)	157 (82.2)
Prospective non‐drinker	14 (3.7)	360 (96.3)	10 (5.5)	173 (94.5)	4 (2.1)	187 (97.9)

**Table 5:** Cross‐tabulation of non‐drinking status by method of ascertainment at phase 11 in the whole sample of self‐identified non‐drinkers (*n* = 374).

	*Life‐grid non‐drinker*		*Prospective non‐drinker*	
*Always a non‐drinker*	*Yes*	*No*	*Yes*	*No*
Yes	42 (44.7)	52 (55.3)	12 (12.8)	82 (87.2)
No	7 (2.5)	273 (97.5)	2 (0.7)	278 (99.3)
Life‐grid non‐drinker				
Yes	–	–	9 (18.4)	40 (81.6)
No	–	–	5 (1.5)	320 (98.46)

*n* (%).

#### Predicting objective health outcome

Table 6 outlines estimates obtained from linear regression models of SBP at phase 11 comparing those who reported consuming six or more drinks in a single occasion on a monthly basis to those who did not (current non‐drinkers and other drinking trajectories not shown). The first column shows that those who did so at the present phase had higher SBP values than those who did not [β 3.08 mmHg, confidence interval (CI) = 1.41, 4.76) after adjusting for age, gender, socio‐economic position and smoking status. The second column uses the decade‐based information obtained retrospectively; when only the current decade is used, the estimate is similar to that obtained with present time information (β 2.73 mmHg, CI = 1.11, 4.35). When groups were created using information from the previous two decades, so that those who also consumed six or more drinks in previous decades represented another category, the effect of current consumption was attenuated slightly (β 2.49 mmHg, CI = –3.11, 8.10) and those with a history of consuming six or more drinks in a single occasion were shown to have higher SBP (β 2.91 mmHg, CI = 1.22, 4.60).

**Table 6:** Mean differences in systolic blood pressure (95% confidence interval) by consuming six or more drinks in a single occasion on a monthly basis using data from the past 12 months, current and previous decades (*n* = 4654).

	*Past 12 months* a	*Current decade* a	*Life course* b
*No*/*never*	*Ref*.	*Ref*.	*Ref*.
Past 12 months/ current decade	3.08 (1.41, 4.76) (*P* < 0.001)	2.73 (1.11, 4.35) (*P* = 0.001)	2.49 (–3.11, 8.10) (*P* = 0.383)
Current decade plus either previous two decades	–	–	2.91 (1.22, 4.60) (*P* = 0.001)

a Non‐drinkers and

b miscellaneous trajectories included as separate categories in the model, but estimates not presented.

## Discussion

### Summary of findings

We found that a retrospective decade based AUDIT‐C grid appears to provide a reliable measure of consumption across the life‐course in older adults. Reliability tended to be greatest for measures relating to frequency of consumption in general as well as frequency of drinking six or more drinks in a single occasion compared to the item relating to number of drinks consumed on a typical drinking day. Current alcohol consumption seemed to influence the accuracy of recall; however, even within strata with hazardous consumption test–retest reliability and correlations between retrospective and prospective measures tended to fall within acceptable ranges. Our findings have implications for both studies that are already in existence (that may not have alcohol data earlier in the life‐course) as well as studies in the planning stage, allowing them to adopt the cross‐sectional cohort 41 approach at baseline to measure alcohol intake earlier in the life‐course.

### Comparison to previous work

Similar to studies examining the test–retest reliability of the LDH questionnaire 14, 15 and validating it using prospective measures 13, 42, we found our AUDIT‐C life‐grid had relatively stable recall and observed high correlations between retrospective and prospective measures.

Like other studies on the stability of self‐reported life‐time abstaining, we found that there were generally large inconsistencies in reporting 7, 19. We found that fewer people identified themselves as life‐time abstainers using the life‐course alcohol grid method than when using a single question regarding life‐time abstention (note that these inconsistencies are within the same individuals). This indicates that single questions on life‐time abstention should be treated cautiously, as they are unlikely to be reliable. However, life‐time abstention using our life‐course grid method was corroborated in only a few cases using prospective data, which further demonstrates the general limitations in identifying life‐time abstainers correctly in alcohol epidemiology 7, 19.

We found that simplified trajectories based on responses to frequency of drinking six or more drinks in a single occasion were related to systolic blood pressure, as in previous studies 17.

### Strengths and limitations

The strengths of our study are the large sample size used for comparing retrospective and prospective measures (previous studies conducting similar work on the LDH have had sample sizes of 1295 13 and 574 42 participants compared to 5980 presently in our study) and that we were able to link prospective information regarding frequency of consumption and usual number of drinks consumed with retrospective measures as a means of validating their utility. An additional strength of our findings is that retrospectively assessed measures of alcohol consumption are associated, in the manner anticipated, with objectively measured systolic blood pressure (improving upon self‐reported measures as used previously in other studies 17).

There are also several shortcomings which need to be addressed and our findings evaluated with them in mind. First, the prospective measures we used to validate retrospective accounts of alcohol consumption across the life‐course do not align perfectly with each other. However, we used polychoric correlations as a means of addressing this issue, and feel confident that although the observed measures are not ideal they resemble the items which they were used to validate closely enough for this not to be a major bias. Closely related to this issue, we lacked prospective information regarding frequency of consuming six or more drinks in a single occasion, and were therefore unable to validate this measure. However, given that test–retest reliability was high for this measure, and the κ coefficients were similar to those observed for the frequency of consumption measure (which we were able to validate), we would argue that this item would probably be as robust—although we welcome future research to confirm or refute this extrapolation.

Another potential source of bias is the age of participants in our study (mean age: 70 years, range = 59–82). One could argue that it is unrealistic to expect those in later life to recollect information on alcohol consumption from up to 60 years earlier. Previous discussions 2, 18 have raised the issue that time‐scale matters with regard to assessing life‐course alcohol consumption retrospectively; however, we return to an argument made in the background section. Expecting people to recall detailed information about their drinking habits (such as daily drinking habits, consumption broken down by beverage type, absolute number of drinking days, etc.) over such a long period is likely to be less reliable than simpler methods, which focus only on core components of drinking behaviour and provide response options for participants to choose from. We believe that data collected using this approach is in keeping with the ethos that retrospective measures of alcohol consumption should be used to gain an overall picture of a participant's drinking history, and not a detailed account 18.

We used data from the Whitehall II cohort of British civil servants, which is not a representative sample of the general population. It is also worth noting that our sample is made up of individuals who have remained in an epidemiological study for ~28 years. This is a form of selection bias 43, which is likely to mean that our sample is no longer representative of the original population from which it was drawn (generally those remaining in the cohort are a healthier subsample 5). However, this bias does not affect the internal consistency of our estimates.

### Directions for future work

In addition to the avenues for future work outlined above, it is important to determine the extent to which decade‐based measures of consumption fail to capture important changes (e.g. moving from high to low or no consumption) across the same 10‐year period and how this shortcoming in the method may influence exposure–outcome associations. While adding this additional level of detail to our life‐course alcohol grid is possibly unnecessary (continuing the philosophy of less is more when it comes to addressing life‐course consumption with retrospective measures), it is worth trying to quantify this bias so that appropriate correction factors may be derived.

Furthermore, it is crucial that the reliability of our adapted life‐course AUDIT‐C is examined in other cultures (and indeed replicated using other UK data sources), as previous studies have shown that there are cultural differences in the retrieval strategies used by individuals across Europe to answer standard questions relating to alcohol consumption 44.

## Conclusion

While prospectively collected data should be preferred, it appears that a decade‐based AUDIT‐C grid seems to provide a reliable measure of consumption across the life‐course in older adults. This method could be used in existing longitudinal studies where alcohol intake earlier in the life‐course is not already measured, included at the baseline of studies in planning to gauge alcohol intake prior to enrolment, or used in cross‐sectional studies.

## Declaration of interests

None.
